# Genomic epidemiology of SARS-CoV-2 during the first four waves in Mozambique

**DOI:** 10.1371/journal.pgph.0001593

**Published:** 2023-03-06

**Authors:** Nalia Ismael, Stephanie van Wyk, Houriiyah Tegally, Jennifer Giandhari, James Emmanuel San, Monika Moir, Sureshnee Pillay, Christian Utpatel, Lavanya Singh, Yeshnee Naidoo, Upasana Ramphal, Nédio Mabunda, Nuro Abílio, Paulo Arnaldo, Joicymara Xavier, Daniel Gyamfi Amoako, Josie Everatt, Yajna Ramphal, Arisha Maharaj, Leonardo de Araujo, Ugochukwu J. Anyaneji, Derek Tshiabuila, Sofia Viegas, Richard Lessells, Susan Engelbrecht, Eduardo Gudo, Ilesh Jani, Stefan Niemann, Eduan Wilkinson, Túlio de Oliveira

**Affiliations:** 1 Instituto Nacional de Saúde (INS), Marracuene, Mozambique; 2 Division of Medical Virology, Faculty of Medicine and Health Sciences, Stellenbosch University, Cape Town, South Africa; 3 Centre for Epidemic Response and Innovation (CERI), School of Data Science and Computational Thinking, Stellenbosch University, Stellenbosch, South Africa; 4 Kwazulu-Natal Research Innovation and Sequencing Platform (KRISP), Nelson R Mandela School of Medicine, University of Kwazulu-Natal, Durban, South Africa; 5 Molecular and Experimental Mycobacteriology, Research Center Borstel, Borstel, Germany; 6 Institute of Agricultural Sciences, Universidade Federal dos Vales do Jequitinhonha e Mucuri, Unaí, Brasil; 7 Centre for Respiratory Diseases and Meningitis, National Institute for Communicable Diseases (NICD) of the National Health Laboratory Service, Johannesburg, South Africa; 8 School of Health Sciences, College of Health Sciences, University of KwaZulu-Natal, KwaZulu-Natal, South Africa; 9 German Center for Infection Research, Partner Site Hamburg-Lübeck-Borstel-Riems, Borstel, Germany; Fundacao Oswaldo Cruz, BRAZIL

## Abstract

Mozambique reported the first case of coronavirus disease 2019 (COVID-19) in March 2020 and it has since spread to all provinces in the country. To investigate the introductions and spread of SARS-CoV-2 in Mozambique, 1 142 whole genome sequences sampled within Mozambique were phylogenetically analyzed against a globally representative set, reflecting the first 25 months of the epidemic. The epidemic in the country was marked by four waves of infection, the first associated with B.1 ancestral lineages, while the Beta, Delta, and Omicron Variants of Concern (VOCs) were responsible for most infections and deaths during the second, third, and fourth waves. Large-scale viral exchanges occurred during the latter three waves and were largely attributed to southern African origins. Not only did the country remain vulnerable to the introductions of new variants but these variants continued to evolve within the borders of the country. Due to the Mozambican health system already under constraint, and paucity of data in Mozambique, there is a need to continue to strengthen and support genomic surveillance in the country as VOCs and Variants of interests (VOIs) are often reported from the southern African region.

## Introduction

The severe acute respiratory syndrome coronavirus 2 (SARS-CoV-2), has since its emergence spread globally. Due to ongoing genome surveillance efforts, ~14 million SARS-CoV-2 full genome sequences are currently publicly available on the web-based platform Global Initiative on Sharing Avian Influenza Data (GISAID) [1]. This large collection of publicly available genome sequences facilitates comparative investigations that provide invaluable insights on SARS-CoV-2 evolution and spread [2, 3]. Comparable to other RNA viruses, SARS-CoV-2 is characterized with rapid rates of mutation and the accumulation of these mutations gave rise to noteworthy lineages with altered viral phenotypic traits. These lineages often increase in frequency within human populations and therefore displace their genetic predecessors [4]. Changes in genetic composition may facilitate the emergence of Variants of Concern (VOCs), to evolve enhanced pathogenicity and transmission capabilities to spread through populations and regions [5–7]. As the pandemic progressed, it was these genetically diverged VOCs that fueled localized and global infectious waves. Such variants are denoted by the World Health Organization (WHO) as VOCs, Variants of Interest (VOIs), or Variants Under Monitoring (VUMs), depending on their frequency, biological characteristics, and potential influence on the pandemic [8].

Genome surveillance allows the detection, reporting, and monitoring of VOCs, VOIs, and VUIs. This information is of crucial importance to determine the potential threats posed by newly emerging SARS-CoV-2 lineages. Genome surveillance allows for informed public health responses and the implementation of disease mitigation strategies. For instance, mutations in these variants were often associated with enhanced transmissibility, disease severity [9], and ability to escape host immune responses, which may influence vaccine efficiency [10]. In this regard, a thorough understanding of the genetic composition, spread and biological features of circulating viruses remains of global importance.

The first case of COVID-19 in Mozambique was reported on 22 March 2020 in Maputo city and the epidemic subsequently spread to all provinces of the country. By April 2022, the continued transmission of the virus resulted in four waves of infection, >225 000 reported cases, and >2 000 deaths. To date, no robust epidemiologic and genomic analyses on the COVID-19 pandemic in Mozambique were conducted. Therefore, we set out to investigate the genetic background of the SARS-CoV-2 epidemic in Mozambique for the first 25 months. We analyzed the SARS-CoV-2 whole-genome sequences from Mozambique available at GISAID until 4th of April 2022 and contrasted it against that of a global subset to describe the VOCs and other unique viral lineages circulating in Mozambique from July 2020 to April 2022. Using this information, we further inferred the estimated number, and timing of viral introductions into Mozambique.

## Material and methods

### SARS-CoV-2 detection and sample selection for genomic analysis

For this study, samples from 1 612 nasopharyngeal and oropharyngeal swabs obtained from routine diagnostic PCR analyses of patients displaying symptoms of COVID-19, collected between July 2020 to April 2022, were suspended in viral transport medium (VTM) and subjected to whole genome sequencing. These samples were collected from all provinces in Mozambique (S1 Table). The collected samples were subjected to SARS-CoV-2 real-time PCR assay on the Abbott m2000 sp/rt system (Abbott Molecular Inc, Germany) targeting the RNA-dependent RNA polymerase (RdRp) and Nucleocapsid (N) genes, according to the manufacturer’s protocol [11]. Samples with a cycle threshold (CT) value below 30 were selected for whole genome sequencing (Oxford Nanopore and Illumina platforms) performed at the Kwazulu-Natal Research Innovation and Sequencing Platform (KRISP) in Durban, South Africa (n = 1 262), Research Center Borstel (RCB), Germany (n = 300), or the National Institute of Communicable Diseases (NICD) in Johannesburg, South Africa (n = 50) (S1 Table, S1 Fig).

### RNA extraction and tiling-based polymerase chain reaction

For the samples submitted to KRISP and NICD, the automated Chemagic 360 instrument was used for RNA extractions using the CMG-1049 kit (Perkin Elmer, Hamburg, Germany) according to the manufacturer’s instructions. Isolated RNA was stored at -80°C. Complementary DNA (cDNA) was synthesized using random hexamer primers and the SuperScript IV reverse transcriptase synthesis kit (Life Technologies), multiplex PCR according to the nCoV-2019 ARTIC network sequencing protocol (https://artic.network/ncov-2019) using the ARTIC amplicon v3 and v4 primer sets and Midnight whole genome sequencing protocols [12, 13]. PCR products were purified using AmpureXP purification beads (Beckman Coulter, High Wycombe, UK) quantified on the Qubit 4.0 (Life Technologies Carlsbad, CA) using the Qubit dsDNA High Sensitivity assay. The purified PCR products were subjected to either Illumina or Oxford Nanopore whole genome sequencing, depending on reagent availability. For samples processed at the RCB, total RNA was extracted with the QIAamp Viral RNA Mini Kit (QIAGEN) from nasopharyngeal/oropharyngeal swabs taken for standard diagnostic testing and tested positive for SARS-CoV-2. cDNA was generated by reverse transcription of total RNA using the LunaScript RT SuperMix Kit (NEB). Further, viral cDNA was amplified using Q5 Hot Start High-Fidelity 2X Master Mix (NEB) and the ARTIC v3 tiled amplicon panel [14] which primers were rebalanced to achieve more even genome coverage (dx.doi.org/10.17504/protocols.io.bibtkann).

### Library preparation and Illumina sequencing

Next generation sequencing was performed using Illumina (Illumina, San Diego, CA, USA) MiSeq and NextSeq 550 platforms. The Nextera Flex DNA library preparation kit, the Nextera CD indexes, and MiSeq Reagent Kit v2 (500 cycles) were used. Libraries were normalized, pooled, and denatured, as described previously [15, 16]. For the NextSeq 550 genome sequencing, library preparation and sequencing were performed using the Illumina COVIDSeq protocol, using IDT for Illumina Nextera UD Indexes for adapter ligation, and a 300-cycle NextSeq 550 HighOutput Kit v2. At RCB, libraries for 2x150bp sequencing on the NextSeq 500 (Illumina) were generated from amplicons using a modified Nextera protocol [17].

### Tiling polymerase chain reaction (PCR) and Oxford nanopore sequencing

Sequencing libraries were generated using the Genomic DNA Sequencing Kit SQK-LSK108 (Oxford Nanopore Technologies). Tiling PCR amplicons were generated as described above. Ligation was carried out using UltraII End Prep Reaction Mix and UltraII End Prep Enzyme Mix and barcoded using the Native Barcoding Kit (Oxford Nanopore Technologies, Oxford, UK). The libraries were cleaned using AmpureXP purification beads (Beckman Coulter, High Wycombe, UK) in a 1:1 ratio and eluted in 15 μl of elution buffer. DNA quantification was performed using the Qubit dsDNA High Sensitivity assay on the Qubit 4.0. Sequencing libraries were loaded onto an R9.4 flow cell, run on an Oxford Nanopore GridION instrument, and data were collected for up to 21 hours.

### Sequence assembly and quality control

Raw reads from Illumina and Nanopore sequencing were assembled using the Genome Detective Coronavirus Typing Tool v133-135 [18, 19]. Raw reads from the Illumina COVIDSeq protocol were assembled using the Exatype NGS SARS-CoV-2 pipeline v1.6.1 (https://sars-cov-2.exatype.com/) [20]. Alternatively, samples sequenced from the Nanopore GridION were assembled with the Arctic-nCoV2019 SARS-CoV-2 assembly pipeline (https://artic.network/ncov-2019/ncov2019-bioinformatics-sop.html). Sequence quality control reports for the assemblies were obtained from Genome Detective, or the ARCTIC pipeline using Next Clade analysis [18, 19, 21]. The sequences were subjected to manual curation. This was done to remove sequencing and assembly errors, sequences with low coverage (<80%), low-quality mutations that included clustered mutations (>3 consecutive mutations), mid-gene stop codons and insertions/deletions that interrupt open reading frames. Regions with clustered mutations and deletions resulting in frameshifts were annotated as gaps and insertions were removed. From the 1 612 samples, the 776 from KRISP, 149 from RCB and 26 from NICD (S1 Fig) resulting in genome assemblies with genome coverage >80%, relative to that of the Wuhan-Hu-1 reference assembly, were deposited on the GISAID (https://www.gisaid.org/) database [1]. For samples generated by RCB, raw reads were analyzed with the SARSCOVseq pipeline (https://github.com/ngs-fzb/SARSCOV2seq). Briefly, the reads were mapped with BWA [20, 22] to the MN908947.3 reference and mappings were refined by INDEL realignment with GATK 3.8 [21, 23]. Amplicon primer were trimmed after alignment and aberrant amplicon reads crossing expected amplicon boundaries were removed with iVar [22, 24], also trimming for base quality 20 in a sliding window of 4 and removing unmapped and < = 30bp reads. The consensus sequence was generated with iVar and an allele frequency cutoff of 0.9, base quality cutoff of 20 and minimum depth of 10 reads. Consensus sequences passed when the sequence identity was > = 0.9 and N-content less than < = 0.05 and were subsequently uploaded to GISAID.

### Epidemiological analysis and reproductive number estimation

The daily estimates of the effective reproductive number (Rₑ estimates) and reported case numbers for Mozambique were plotted for the period of 1 April 2020 to 22 April 2022. The number of daily cases of COVID-19 and deaths were accessed from the COVID-19 Data Repository by the Center for Systems Science and Engineering at John Hopkins University [23, 25]. Daily estimates of the Rₑ number (Median Rₑ with 95% Highest Posterior Density (HPD) intervals were retrieved from COVID-19 Rₑ (https://github.com/covid-19-Re/dailyRe-Data) [24, 26] Rₑ indicated the average number of secondary infections and serves as a criterion to measure the extent and progression of the COVID-19 pandemic. For these analyses, an Rₑ value >1 represented a growing epidemic, and Rₑ values <1 indicate a reduction in disease transmission [27].

To investigate the generation of genomic sequencing relative to the progression of the epidemic, the number of sequenced SARS-CoV-2 genome assemblies published between 1 April 2020 to 22 April 2022 were retrieved from GISAID (https://www.gisaid.org/). The dataset was accessed on 22 April 2022 and reflected >80% query coverage relative to that of the Wuhan-Hu-1 reference assembly. The number of SARS-CoV-2 genomes produced per province was plotted to illustrate the spatial distribution of genomic surveillance across Mozambique, which included information on the origin and province from which the sample was obtained. The temporal progression of SARS-CoV-2 lineages was represented by the genome count per lineage over the sampled date. Figures were generated using R Studio (R Studio Team 2020).

### Phylogenetic analyses

The sampling strategy for phylogenetic analyses aimed to include SARS-CoV-2 whole-genome sequences reflecting global and Southern African viral diversity. The dataset consisted of 19 500 sequences. Of these sequences, a total of 1 142 sequences were sampled from Mozambique and retrieved from the GISAID database (date of access: 22 April 2022). Additionally, global, and African representative datasets were included in these analyses and accessed through the NextStrain platform (https://nextstrain.org/ncov/gisaid/global). These were supplemented with sequences obtained from samples collected in other countries but labeled Mozambique as the country of exposure. Due to the lack of sequence information from Tanzania all global sequences with Tanzania as the country of exposure were included.

The C.1. and the associated sub-lineage C.1.1, which is unique to southern Africa [28], were investigated in greater detail to gain insight into virus transmission in the country. To this end, all available C.1.1 Mozambican sequences, and C.1 sequences from neighboring countries (n = 137) were included in the analyses. South Africa sequences included those that were sampled from provinces that border Mozambique, i.e., Limpopo (n = 379), Mpumalanga (n = 380), and KwaZulu-Natal (n = 925). For comparative purposes, Gauteng sequences (n = 1 412) were included in the reference set. Additional South African references from other provinces were permitted as part of the African or global builds of NextStrain.

The SARS-CoV-2 whole-genome sequences were aligned with the use of NextAlign [21] and curated manually to ensure accurate codon alignment in comparison to that of the Wuhan reference strain using Geneious Prime version 2021.1.1. The tree topology was inferred by performing maximum likelihood (ML) analyses and was implemented using FastTree [29]. For these analyses a General Time Reversible model was applied as the nucleotide substitution model, and ML analyses were implemented on 1 000 bootstrap replicates to infer the branching structure of the resulting phylogeny. For these analyses, annotations to the dataset were assigned according to the geographic origin of each strain using Phylotype, and standard parameters were applied (size ≥5, size/different ≥1, persistence ≥1, nodes which ML bootstrap values were ≥70 were considered, tested for ACCTRAN optimization, and 1000 iterations of shuffling procedures).

Due to the higher mutation rate of the Omicron VOC we opted to perform non-Omicron and Omicron time-scaled phylogenies. Briefly, the original alignment was split into non-Omicron and Omicron subsets. ML tree topologies were inferred using FastTree as described above. Following tree reconstruction, a root-to-tip regression of each tree was performed using the clock package extension of TreeTime and the estimated inferred rate of evolution was determined. Subsequently, the ML-trees were transformed into time-calibrated phylogenies with the application of the inferred mutation rates. Outlier sequences were removed with the use of the Ape package in R [30]. For the non-Omicron subset, a rate of 8x10^-4^ substitutions/site/year was applied. For the Omicron dataset a rate of 6x10^-4^ substitutions/site/year was applied as was identified using the clock functionality of TreeTime [31]. Dated phylogenies along with their associated metadata were then used to map discrete country locations onto the trees and to infer country locations for internal nodes in the trees. Finally, an in-house python script was used to count the number of state changes from the root towards the tips of the trees to infer the number of viral introductions and exports between Mozambique and the rest of the world. To gauge the uncertainty in our inferred estimates the process was repeated on 10-bootstrap tree replicates for both the Omicron and non-Omicron subsets. The results represented an aggregate of the Omicron and non-Omicron datasets.

### Ethical approval

This study was approved by the National Committee for Bioethics in Health (CNBS) of Mozambique (Reference number 285/CNBS/20 and 24/CNBS/21) and the Biomedical Research Ethics Committee (BREC) of the University of KwaZulu-Natal (BREC/00001510/2020).

At the time of sample collection, the mobile phone number of these individuals were registered on the Laboratory Test Request form for result dissemination. Since most of the mobile numbers of these participants were available on the database, oral informed consent was obtained. The consenting process was conducted through a mobile phone for all potential study participants that had their samples stored.

## Results

### SARS-CoV-2 epidemiology dynamics in Mozambique

**Fig 1:**
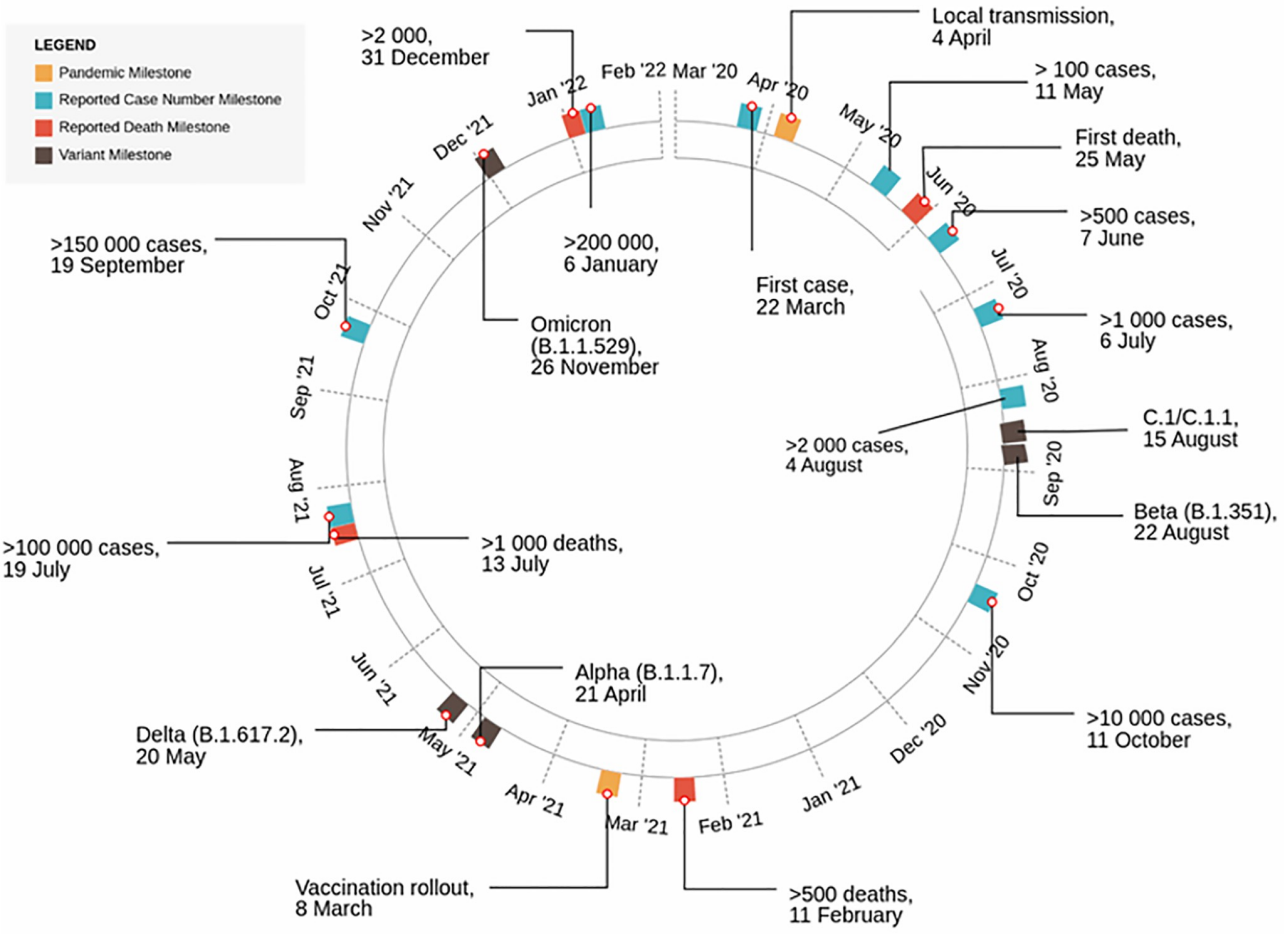
Schematic representation of the progression and milestones occurring during the COVID-19 pandemic in Mozambique (March 2020- April 2022). The number of reported COVID-19 cases milestones were illustrated in blue, the total deaths milestones are illustrated in red, the first detection of new variants are illustrated in black, and other key milestones such as local transmission and vaccination rollout are illustrated in orange.

**Fig 2:**
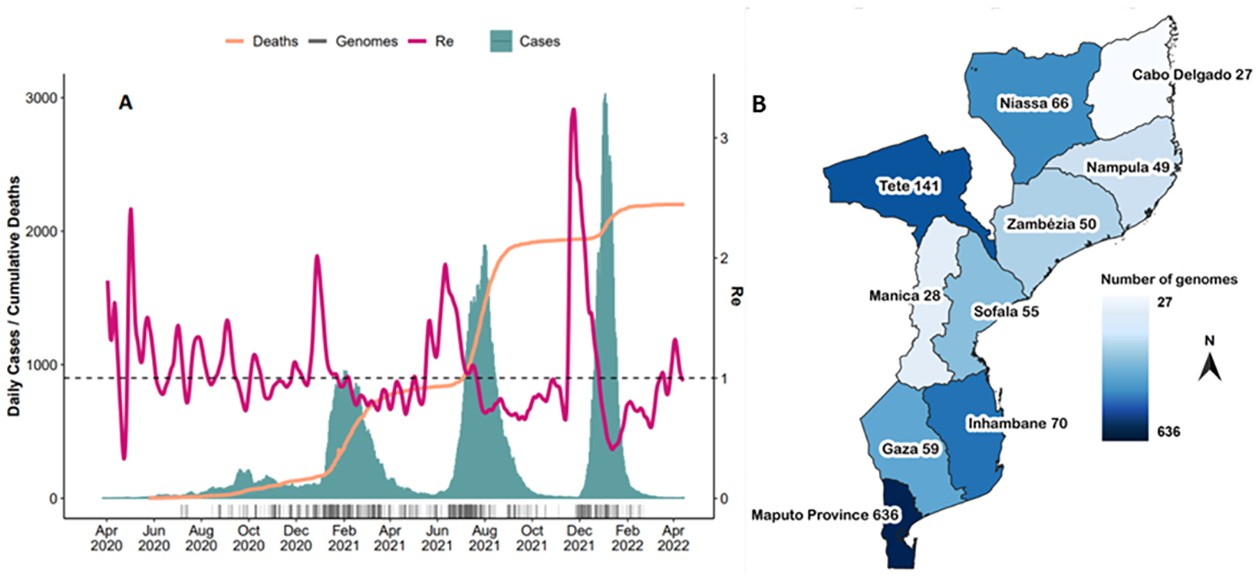
SARS-CoV-2 epidemiological dynamics in Mozambique. (A) Schematic illustration of genomic and epidemiological data. Changes in the daily reported COVID-19 cases are indicated in blue, the cumulative death count associated with COVID-19 disease in orange, and the estimated Re values are indicated in purple. (B) Geographic map of Mozambique illustrating the origin and count genome sequences generated in this study (The map for Mozambique was accessed using the software packages used for mapping, GADM, https://gadm.org/download_country.html). Genome sequences without information on the province from which it originated were omitted from this graph.

The first case of COVID-19 in Mozambique was recorded on 22 March 2020 by a traveler returning from the United Kingdom (Fig 1). On 30 March 2020, the government declared a state of emergency. Pandemic mitigation strategies included the closure of schools, cancellation of most social, cultural, and sports events, restriction on public gatherings, mandatory use of facial masks, night curfew, social distancing, and implementation of travel restrictions. In total Mozambique has endured four waves of infection between March 2020 and April 2022 (Fig 2A). The country experienced an extended first wave with a relatively low count in reported cases, presumably due to the public health measures implemented at the time. The first infectious wave lasted from June to November 2020, with an estimated ~11 000 cases and ~80 deaths recorded over this time. The second wave started in January 2021 and lasted until April 2021, recording ~20 000 cases and ~200 deaths, the third wave started June 2021 and lasted until September of 2021, causing ~70 000 cases and ~1 000 deaths, while the fourth wave started in December 2021 and lasted until January 2022, resulting in ~73 000 cases and ~250 deaths. During March of 2021 the national COVID-19 vaccination campaign was launched.

As expected, the country wide Rₑ values fluctuated during the progression of the pandemic in tandem with each pandemic wave (Fig 2A). During April 2020 an Rₑ value of less than 1 indicated a low pandemic progression and reflected the pandemic mitigation strategies applied in Mozambique at the time. However, the Rₑ value increased to approximately 2 in May 2020, which suggested an increase in transmission during this time.

### SARS-CoV-2 genomes and lineages

**Fig 3:**
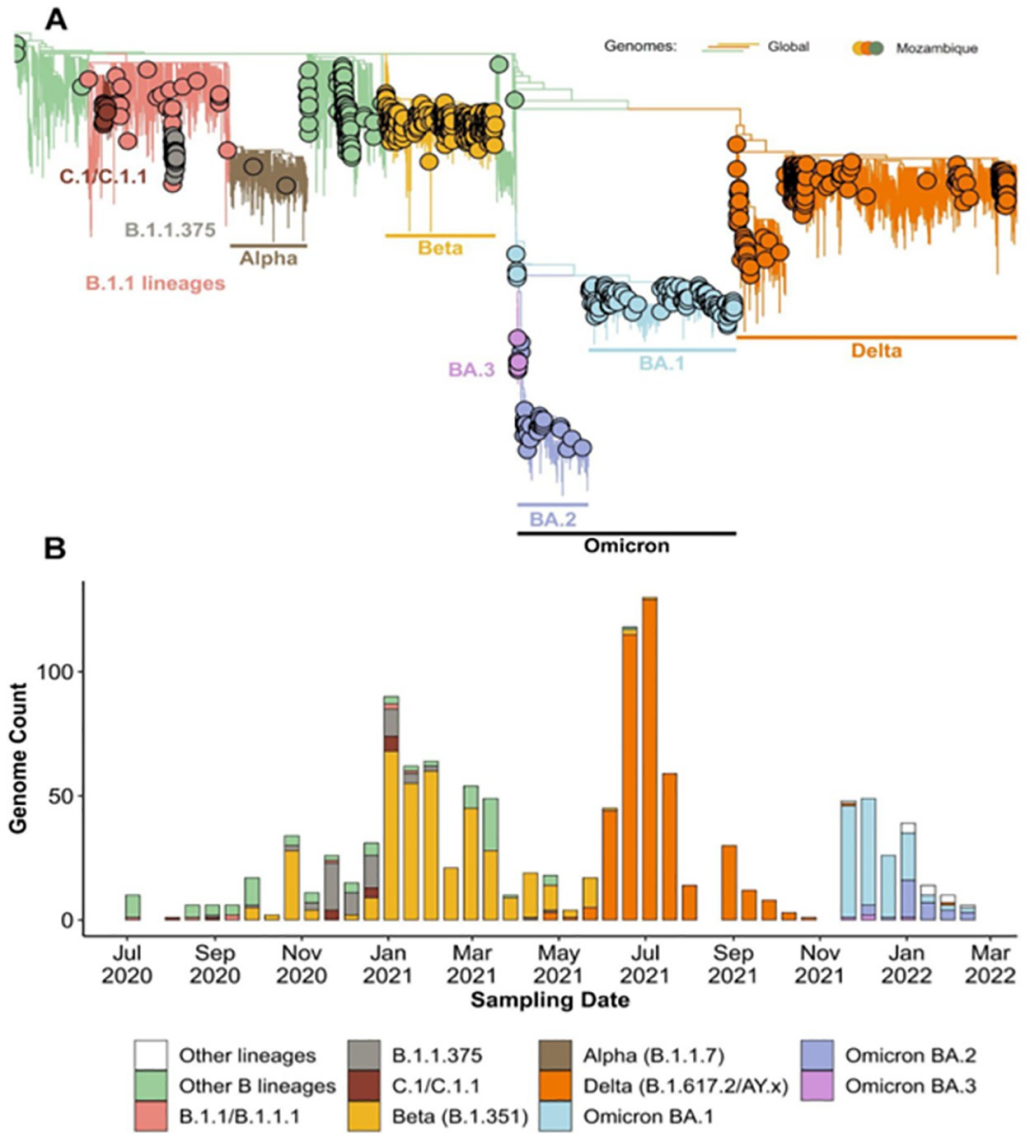
Lineage and phylogenetic analysis. (A) Phylogenetic tree of SARS-CoV-2 sequences representing global and Mozambique subsets during the first 25 months of the pandemic. Taxa obtained from Mozambique are illustrated with circles and the lines represent taxa from the global dataset. (B) Lineage through time plots illustrate the change in genetic diversity of sequenced genomes sampled in Mozambique throughout the study period.

By 22 April 2022, 1 142 genomes from all 10 provinces in Mozambique were deposited to GISAID (S2 Table) and reflected those that were sampled between July 2020 and February 2022 (Fig 2B). Of these samples, 951 whole-genome sequence assemblies were generated for this study, and an additional 191 genome sequences were accessed through GISAID (S1 Fig). These assemblies reflected an average assembly coverage of 96% compared to that of the Wuhan references strain. All provinces were represented in this dataset with more than half sampled in the Maputo province. This province also reported the highest number of cases. More samples were submitted for genome sequencing as case numbers increased. In the interest to maintain effective genome surveillance, more sequences were submitted for genome sequencing during times when neighboring countries reported a higher prevalence of VOIs and VOCs. The most sequenced isolates were classified as Beta (B.1.351, 363 genomes; 42.61%), Delta (B.1.617.2; 259 genomes; 30.4%), and Omicron (B.1.1.529; 177 genomes) variants. Notably, these variants dominated the epidemic in Mozambique during the second, third, and fourth waves, respectively (Figs 1 and 3), comparable to epidemic profiles in other southern African countries [28, 32].

Samples isolated during May and June 2021 indicated the presence of the Alpha and Delta variant in Mozambique that were coupled with an increase of local transmission and deaths. In July 2021, Mozambique reported >100 000 cases, which increased to 150 000 in September, and >200 000 in December of 2021. The Omicron variant, first detected in South Africa, was reported in Mozambique in late November 2021.

Temporal analyses of SARS-CoV-2 indicated a combination of genetically distinct viruses circulating in Mozambique and reflected a similar composition to that of neighboring South Africa (Fig 3B). During the first wave, a low number of genomes sampled in Mozambique were generated representing the initial onset of the epidemic The B.1, B.1.1, and C.1 lineages were circulating during the first epidemic wave. During the second infectious wave, the Beta variant was the dominant lineage; however, C.1, C.1.1, B.1.1, B1.1.1, and B1.1.375 were also detected in low numbers. The Beta variant, which was first detected in October 2020 in 13 samples from Maputo, displaced many of these lineages and remained the dominating circulating variant during the second Mozambican wave which peaked in January of 2021. The Delta variant displaced the Beta variant in the subsequent third wave. For the fourth wave the dominating variant was Omicron which was initially detected in Maputo during November 2021. These included the BA.1, BA.2, and BA.3 sub-lineages. It should be noted that the Delta variant was also circulating in Mozambique during the fourth infectious wave, albeit at low prevalence. Besides for the C.1.1 lineage, no other Mozambique-specific variants were detected.

For the C.1.1 lineage, 12 Mozambican genome sequences were sampled during November (n = 2) and December (n = 5) of 2020, and January 2021 (n = 5) from the Maputo (n = 7) and Inhambane (n = 5) provinces of Mozambique (S3 Table). The C1.1 lineage was detected at low prevalence and represented 1% of Mozambican sequences generated during the study period. Moreover, C1.1 occurred during the first infectious wave and did not reach dominance in Mozambique or elsewhere. The C.1.1 lineage emerged during the time that the Beta variant was dominant in Mozambique, and C.1.1 was subsequently outcompeted by the Beta variant. The C1.1 lineage further represented a monophyletic clade descending from its C.1 progenitor. The C.1.1 showed 12–18 non-synonymous mutations compared to the Wuhan reference strain (S3 Table). Of these mutations, the S477N and D614G mutations for the Spike Open Reading Frame (ORF), and the R203K/G204R for the nucleocapsid ORF were present in all Mozambique C.1.1 sequences. For the Spike ORF, the A6886S and M1237I mutations co-occurred and were prominent (58%; n = 7) in the C.1.1 lineage.

### Phylogenetic analyses

The results of the phylogenetic analyses show multiple and independent introductions of SARS-CoV-2 into Mozambique during the first 25 months of the pandemic (Fig 3A and 3B). For 2020, these included the B.1.1, B.1.1.1, B.1.1.375, and C.1 variants. The C.1 variant was introduced to Mozambique during July 2020 most likely from South Africa where it was first identified [33]. Following this introduction, the C.1 lineage underwent genetic diversification and continued to evolve and was later classified as a sub-lineage called C.1.1 (Fig 3B) as previously described [28]. The Beta variant had multiple introductions and community transmission were inferred during October of 2020. The Alpha variant was most likely introduced at least twice in April 2021 without wide-spread community transmission. The Alpha variant did not circulate in high prevalence most likely due to the high prevalence of the Beta variant circulating in Mozambique at the time. Similarly, the Delta variant showed multiple independent introductions during June and July of 2021. These introductions were inferred to have originated from southern Africa and not from India. Moreover, the shared recent phylogenetic history of the Mozambican Delta samples suggests that at least four localized outbreaks occurred in Mozambique. These outbreaks initially occurred in Tete, the central region of the country with an increase in cases in the capital city of Maputo, and it subsequently spread throughout the country. In late November and December 2021, the Omicron variant was introduced on multiple occasions and was subjected to localized transmission. The Omicron sub-variants BA.1, BA.2, and BA.3 were similarly introduced, and localized transmissions were inferred during 2022.

### Import and export analysis of Mozambican SARS-CoV-2 samples

**Fig 4:**
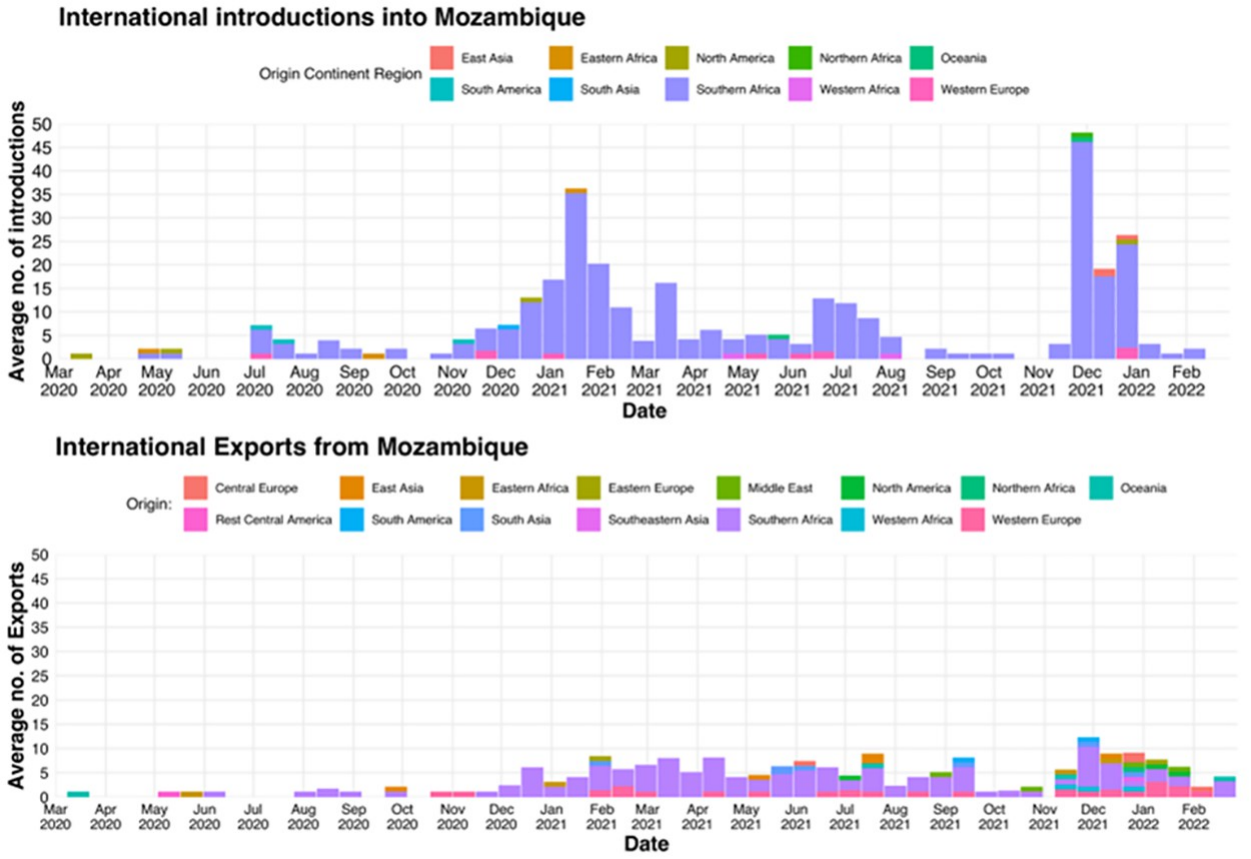
SARS-CoV-2 import and export events inferred for Mozambique. The bar charts illustrate the average number of SARS-CoV-2 introduction events (top), and the average number of export events (bottom).

The SARS-CoV-2 virus was introduced into Mozambique on numerous occasions (Fig 4). Although the first confirmed case that was officially reported on the 22nd March 2020 was imported from the United Kingdom, the analysis revealed that first introductions originated from North America in March 2020. During the 25-month study period, SARS-CoV-2 introductions originated from East Asia, Eastern Africa, North America, Northern Africa, Oceania, South America, South Asia, Southern Africa, Western Africa, and Western Europe. Notably, the majority of introductions originated from Southern Africa (n = 98). November 2021 had the highest count of introductions (n = 66), originating from Southern Africa (n = 60). An increase in inferred introduction events also occurred during December 2020 to February 2021 (Fig 4). The introductions originated from Eastern- (n = 1) and Southern Africa (n = 101), and Western Europe (n = 1).

Phylogenetic analyses and import/export inference showed that numerous export events of SARS-CoV-2 occurred from within the borders of Mozambique to Central Europe, East Asia, Eastern Africa. Eastern Europe, the Middle East, North America, Northern Africa, Oceania, Central America, South America, South Asia, Southern Africa, Western Africa, and Western Europe. The first of which occurred in March of 2020 and was exported to North America. Isolated exportation events occurred during 2020, and included geographic destinations such as Central America, Eastern Africa, Southern Africa and East Asia. These events steadily increased in late December 2020 and clusters of export events occurred until September 2021 and resumed in November 2021 to the end of February 2022 (Fig 4B). Southern Africa received the majority of the viral exportations from Mozambique.

## Discussion

Epidemiological analyses indicated that as of April 2022 Mozambique endured four waves of COVID-19 infection, causing >225 000 reported cases and the death of >2 170 people during the first 25 months. Mozambique has experienced fourth waves which were associated with higher case numbers, but lowered deaths. These pandemic waves were comparable to that of neighboring countries [28, 32, 34], however Mozambique reported substantially less deaths. A thorough understanding of the pandemic progression during the first 25 months provided key insights on the pandemic progression in Mozambique, such as detection and monitoring the frequency, and the international dissemination of VOCs and VOIs into and from Mozambique. Additionally, this work illustrated the continuous evolution of the virus within Mozambique. This knowledge can be applied to navigate the pandemic going forward. Lower COVID-19 mortality rates were also observed among other African countries when compared with higher-income countries [35].

Phylogenetic inference indicated multiple introductions of SARS-CoV-2 variants and VOCs into Mozambique. Four of the five VOCs lineages were prevalent and circulating within the country during the COVID-19 pandemic. The VOC Gamma was not detected in Mozambican samples investigated, however other variants including the B.1 ancestral lineages, the C.1, and C.1.1 lineages were present in notable frequencies. The second, third, and fourth waves were fueled by the Beta, Delta, and the Omicron VOCs, respectively. However, the Alpha variant was also detected in low frequencies during this time. Phylogenetic inference indicated that the C.1 variant, originating from South Africa [28, 33], was introduced into Mozambique. Following localized transmission, the C.1 variant diversified into the sub-lineage C.1.1. These findings agree with previous phylogeographic reconstruction that showed that this lineage was first detected in Johannesburg, South Africa [28, 33], was introduced to Mozambique, where it continued to evolve and established the C.1.1 lineage. Previous studies showed that the C.1.1 sub-lineage acquired spike protein mutations S477N (Ser477→Asn), A688S (Ala688→Ser), and M1237I (Met1237→Ile). Comparable to the B.1.525 lineage, C.1.1 also acquired the Q52R and A67V mutations [28, 33]. These mutations may underlie evolution towards enhanced transmissibility and explain its prevalence and persistence in Mozambique.

The geographic location and elongated shape of the country coupled with numerous neighboring countries, including Zimbabwe, Malawi, Zambia, and Tanzania leaves Mozambique vulnerable to the introduction of VOCs and VOIs. High volumes of travel for trade and tourism purposes between the Southern and Central ports of Mozambique, and that of the neighboring countries, may have facilitated the spread of SARS-CoV-2 Indeed, this study reports an epidemiological linkage between Mozambique and its neighboring countries. Epidemiological and genetic data show interesting parallels between Mozambique and South Africa [28], the neighboring country to the South. Due to its proximity to Mozambique, porous borders, and high volumes of commercial trade between these countries, Mozambique remains vulnerable to importation of VOCs and VOIs originating from South Africa. In support of this view, the timing of infectious waves and changes in the genetic frequency of variants in both countries, indicate that epidemiology dynamics of these countries were linked [28, 33, 36]. Most imports were attributed to South Africa because it has the highest testing rates and available genomic surveillance data. Therefore, the epidemiological linkage between Mozambique and other neighboring countries apart from South Africa may have occurred but as a result of limited testing and associated genome surveillance, may not have been detected in our analyses. Despite these limitations, our analyses show that variants such as the C.1 and C1.1 lineages, and VOCs, such as Alpha, Beta, and Delta, were mostly introduced from South Africa during this study period. Previous investigations indicated initial introduction into Mozambique originated from European and North American travelers [34, 37]. Notably, when travel restrictions were implemented during 2020, fewer introduction events were inferred. Besides the importation of COVID-19 cases from other countries, the virus was disseminated from Mozambique to other parts of the world via travel. Indeed, this study noted numerous international export events originating from Mozambique. In agreement with these findings, a recent event of an Italian traveler returning from Mozambique, exported the Omicron variant into Italy [35, 38]. Taken together, the results of this study, and that of others [28, 34, 37], suggest that SARS-CoV-2 variants were introduced, circulated, continued to evolve in Mozambique, and that lineages unique to Mozambique can potentially be disseminated to other countries.

The findings of this study emphasized the global importance of effective genome surveillance in Mozambique. Of the total 1 142 Mozambican genome sequences available on GISAID, 951 assemblies of SARS-CoV-2 were generated for this study. The majority of genomic data generated indicate that the Beta and Delta variants were the most prevailing isolates sequenced in Mozambique during the study period. Most samples originated from the city of Maputo and Tete. Very few samples were received from the Northern provinces such as Cabo Delgado and Nampula. This may possibly be explained by reduced number in sample submissions for sequencing, coupled with reduced sample quality due to an ineffective sample referral systems and courier pipelines implementing cold chain management. Considering that VOCs and VOIs are often reported from Southern African countries [35, 39, 40] these findings continue to have implications on both local and global response efforts. This disparity in data from these northern provinces creates a so-called “blind spot” in the ongoing genomic surveillance efforts. This finding is particularly concerning as Tanzania, the northern neighboring country, deposited negligible counts of genomic data to open access repositories, limiting the effectiveness of ongoing genomic surveillance efforts in this region. Moreover, the findings of this study indicated continued evolution of SARS-CoV-2 variants within Mozambique and Africa, and thus reiterates the need for scientific transparency and data availability across southern and eastern Africa. In other words, limited testing, sampling, and genomic surveillance in these regions may influence global health matters and threaten pandemic mitigation efforts.

Further limitations of the data generated in this study included the restricted access to in-country genome reagents and trained personnel for genomic sequencing during the early stages of the pandemic. Therefore, Mozambique had to rely on external laboratories for sequencing. Sequencing capacity in Mozambique was established at the National Institute of Health during 2021, making Mozambique less dependent on international laboratories for COVID-19 and future pathogen surveillance. Even though testing and genome surveillance capacity in Mozambique improved substantially during the study period, the possibility of earlier breakouts and introduction events may not have been detected with the available information in these analyses. Moreover, reduced testing in neighboring countries such as Zimbabwe, Malawi, and Zambia, limits the informativeness of our analyses. With fewer samples available for genome sequencing, information on localized outbreaks and introductions of variants remains limited. Therefore, these results may be more biased to detecting introductions originating from South Africa. For instance, the relationship between neighboring countries besides South Africa and Mozambique were also characterized with high volumes of trade, porous borders, and limited genome surveillance and viral introduction events would thus not be observed.

## Conclusions

Our results provide a comprehensive view of the genomic epidemiology of SARS-CoV-2during the first 25 months into the pandemic in Mozambique, and highlighting the prevalence of VOCs Beta, Delta, and Omicron during the second, third, and fourth waves. Data from this study shows that first imports and exports of SARS-CoV-2 in Mozambique were from South Africa and to North America, both in March 2020. Additionally, a large number of viral exchanges occurred between South Africa and Mozambique, demonstrating the manner that viral spread can be affected by the trade and commerce among two countries. Altogether, this study emphasizes the importance of continued genomic surveillance of currently circulating variants in Mozambique to better inform policies and strategies to fight against COVID-19 and future pandemics.

## Supporting information

S1 Fig Summary of nasopharyngeal or oropharyngeal swabs collected in Mozambique during the study period that were subjected to whole-genome sequencing, quality control, and submission to GISAID (see text for details). (TIF)

S1 Table Summary of the demographic data and metadata of the samples that tested positive for SARS-CoV-2 and subjected to whole genome sequencing. (XLSX)

S2 Table Summary and metadata for genome assemblies submitted to the GISAID platform. (XLSX)

S3 Table List of the 12 SARS-CoV-2 C1.1 lineage whole-genome assemblies and amino acid changes generated during this study. (XLSX)

S4 Table GISAID contributions and acknowledgeements for SARS-CoV-2 genome assemblies accessed this study. (XLSX)
