# Supplementary figures and images for: Genomic epidemiology of SARS-CoV-2 during the first four waves in Mozambique
Source: PLOS Glob Public Health. 2023 Mar 6;3(3):e0001593. doi: 10.1371/journal.pgph.0001593 (PMC10021167; doi:10.1371/journal.pgph.0001593)

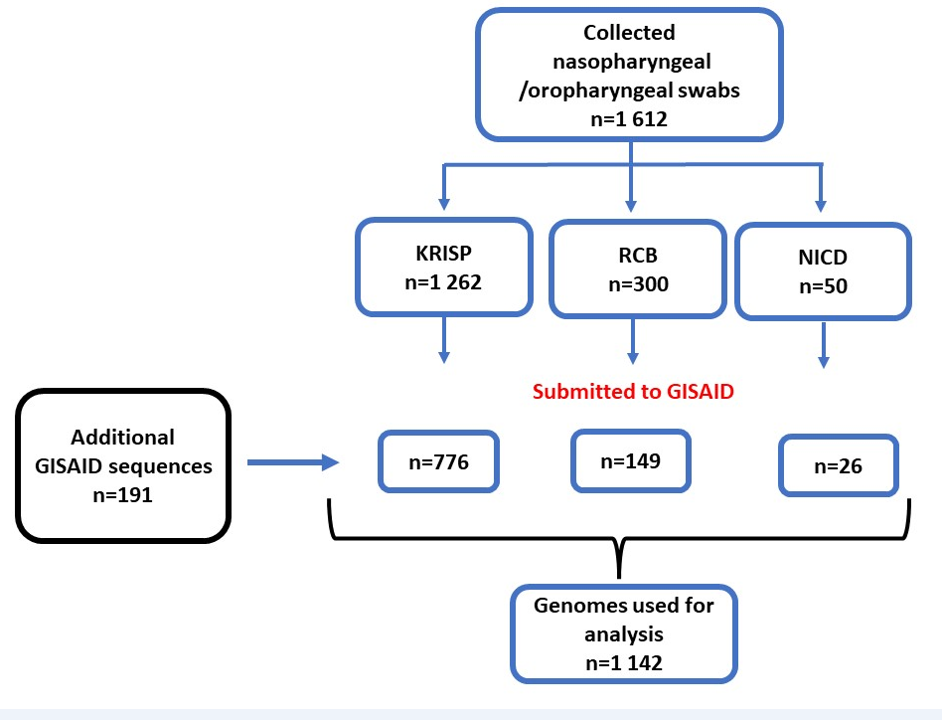

Supplement: S1 Fig — (TIF) [file pgph.0001593.s001.tif]
